# 508. Cabotegravir Long-Acting for Pre-Exposure Prophylaxis (PrEP): Real World Data on On-Time Dosing, HIV Testing and HIV Acquisition from the OPERA Cohort

**DOI:** 10.1093/ofid/ofae631.160

**Published:** 2025-01-29

**Authors:** Anthony M Mills, Laurence Brunet, Kevin R Frost, Ricky Hsu, Gerald Pierone, Michael G Sension, Philip C Lackey, Karam Mounzer, Michael B Wohlfeiler, Jennifer S Fusco, Carolyn A Brown, Vani Vannappagari, Michael Aboud, Piotr Budnik, Gregory P Fusco

**Affiliations:** Men's Health Foundation, Los Angeles, CA, United States(LAX-Los Angeles International Airport), California; Epividian, Inc., Durham, North Carolina; amfAR, The Foundation for AIDS Research, New York, New York; AHF/ NYU Langone Medical Center, New York, NY; Whole Family Health Center, Vero Beach, FL; can community health, Miami Beach, FL; Wake Forest University School of Medicine, Winston Salem, North Carolina; Philadelphia Fight Community Health Centers, Philadelphia, Pennsylvania; AIDS Healthcare Foundation, Miami Beach, Florida; Epividian, Inc., Durham, North Carolina; ViiV Healthcare, Atlanta, Georgia; ViiV Healthcare, Atlanta, Georgia; ViiV Healthcare, Atlanta, Georgia; ViiV Healthcare, Atlanta, Georgia; Epividian, Inc., Durham, North Carolina

## Abstract

**Background:**

Cabotegravir long-acting (CAB LA) pre-exposure prophylaxis (PrEP) consists of 2 initiation injections given 1 month apart, followed by a continuation injection every 2 months, with HIV testing before each injection. We compared characteristics of CAB LA and oral PrEP users and described patterns of CAB LA dosing and HIV testing.

**Figure:**
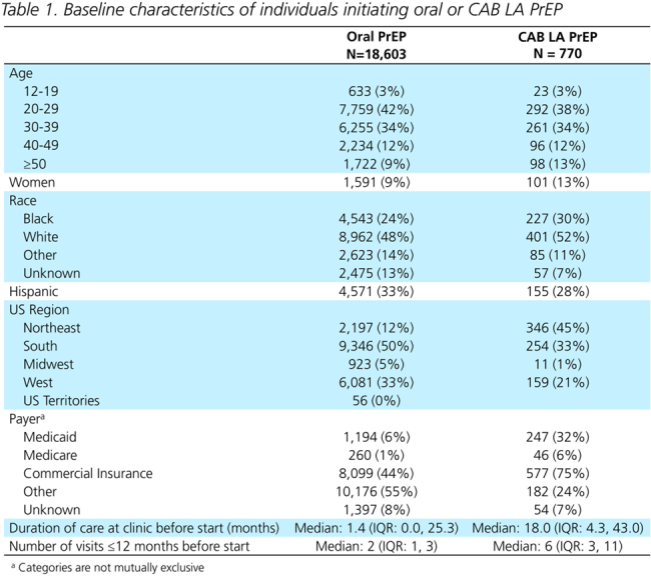

**Methods:**

All individuals ≥12 years old with a new oral PrEP or ≥1 CAB LA injection from 21Dec2021-30Jun2023 in the OPERA cohort were included. CAB LA initiation was completed if the first 2 initiation injections were received ≤60 days apart. Patterns of injections were described in complete initiators as on-time (1 month after the first or 2 months after subsequent injections, ±7 days), delayed ( >7 days after the target), requiring re-initiation per label (≥91 days without injection), or ≥2 injection targets missed (≥128 days without injection). HIV testing ≤1 week before/at each injection and cases of HIV acquisition were described.

**Figure:**
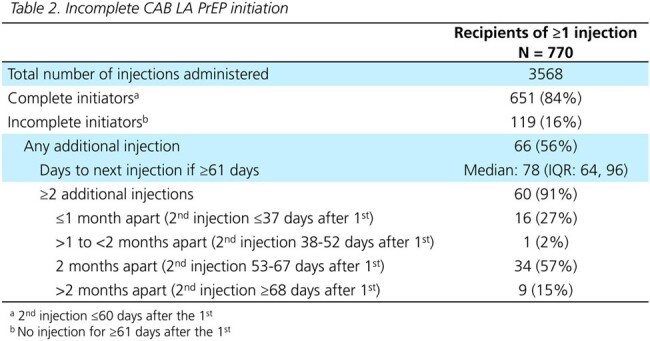

**Results:**

The 770 CAB LA users were more likely to be women, on Medicaid, and have a longer history of care at the clinic than the 18603 new oral PrEP users (Table 1). Of 119 (16%) incomplete initiators, 56% received ≥1 additional injection (Table 2). Most complete initiators received all injections on-time (68%). Of the 206 complete initiators with any delayed injections, 50% received their injection ≤1 week late. A third went ≥91 days and 20% went ≥128 days without an injection (Table 3). Only 66% received an HIV test before the first injection, and 47% before each subsequent injection. Two cases of HIV acquisition were observed among CAB LA PrEP users (Fig 1).

**Figure:**
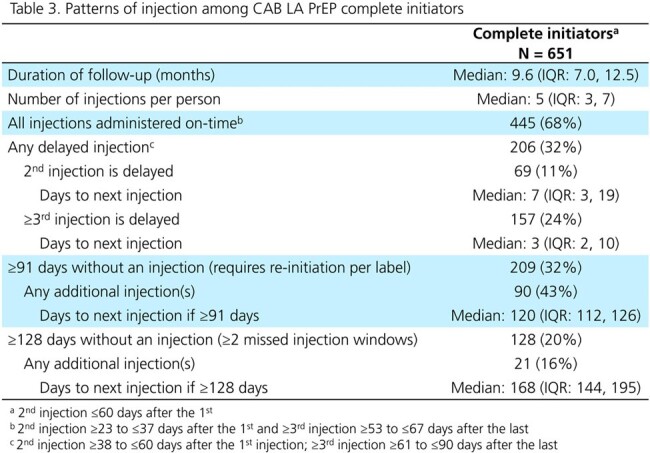

**Conclusion:**

In this large real-world US cohort of 770 individuals receiving 3568 CAB LA injections, while injection delays were observed, most were short. The presence of additional injections after long delays requiring re-initiation suggests ongoing interest in long-acting prevention. No information was available on risk perception or changes in need for PrEP, which could influence patterns of PrEP use. While oral bridging or on-demand PrEP may have been used, it could not be assessed. HIV testing was not conducted as frequently as recommended by the FDA, and the two HIV cases identified could not be linked to CAB LA PrEP use due to its prior discontinuation or to the lack of testing.

**Figure:**
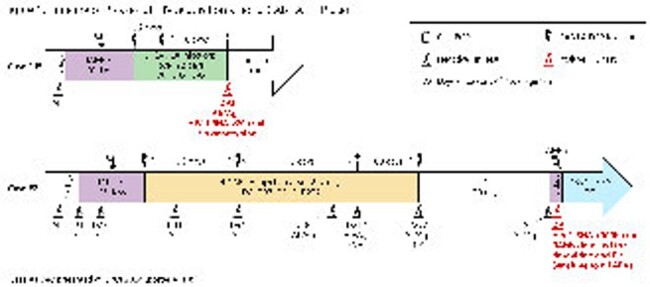

**Disclosures:**

**Anthony M. Mills, MD**, Abbott: Grant/Research Support|Emit Bio: Grant/Research Support|Gilead: Advisor/Consultant|Gilead: Grant/Research Support|Merck: Advisor/Consultant|Merck: Grant/Research Support|ViiV Healthcare: Advisor/Consultant|ViiV Healthcare: Grant/Research Support **Laurence Brunet, PhD**, EMD Serono: Research support to my employer|Gilead Sciences: Research support to my employer|Merck & Co.: Research support to my employer|TheraTechnologies: Research support to my employer|ViiV Healthcare: Research support to my employer **Gerald Pierone, Jr., MD**, GSK: Grant/Research Support|VIIV: Grant/Research Support **Michael G. Sension, MD**, Gilead: Grant/Research Support|Gilead: Honoraria|Viiv: Honoraria **Karam Mounzer, MD**, Epividian: Board Member|Epividian: Honoraria|GS: Advisor/Consultant|GS: Grant/Research Support|GS: Honoraria|Merck: Advisor/Consultant|THERA Technologies: Grant/Research Support|ViiV healthcare: Advisor/Consultant|ViiV healthcare: Grant/Research Support|ViiV healthcare: Honoraria **Jennifer S. Fusco, BS**, EMD Serono: Research support to my employer|Gilead Sciences: Research support to my employer|Merck & Co.: Research support to my employer|TheraTechnologies: Research support to my employer|ViiV Healthcare: Research support to my employer **Carolyn A. Brown, MSPH, PhD**, ViiV Healthcare/GSK: Stocks/Bonds (Public Company) **Vani Vannappagari, MBBS, MPH, PhD**, GSK: Stocks/Bonds (Public Company)|ViiV Healthcare: Full time Employee|ViiV Healthcare: Stocks/Bonds (Public Company) **Piotr Budnik, MBBCh FCP(SA)**, GSK: Stocks/Bonds (Public Company)|ViiV Healthcare: Employee **Gregory P. Fusco, MD, MPH**, EMD Serono: Research support to my employer|Gilead Sciences: Research support to my employer|Merck & Co.: Research support to my employer|TheraTechnologies: Research support to my employer|ViiV Healthcare: Research support to my employer

